# Endoscopic Transnasal Approaches to Petrous Apex

**DOI:** 10.3389/fsurg.2022.903578

**Published:** 2022-05-16

**Authors:** Alexander Kovalev, Rinat Sufianov, Daniel Prevedello, Luís Borba, Luciano Mastronardi, Tatiana Ilyasova, Roy Thomas Daniel, Mahmoud Messerer, Marcio Rassi, Guang Zhang

**Affiliations:** ^1^ Department of neurooncology, Federal Center of Neurosurgery, Tyumen, Russia; ^2^ Department of Neurosurgery, Sechenov First Moscow State Medical University (Sechenov University), Moscow, Russia; ^3^ Department of Neurological Surgery, The Ohio State University, Columbus, OH, United States; ^4^ Department of Neurosurgery, Federal University of Paraná, Curitiba, Brazil; ^5^ Division of Neurosurgery, San Filippo Neri Hospital, Roma, Italy; ^6^ Department of Internal Diseases, Bashkir State Medical University, Ufa, Republic of Bashkortostan, Russia; ^7^ Department of Neurosurgery, University Hospital of Lausanne, Lausanne, Switzerland; ^8^ Department of Neurosurgery, A.C. Camargo Cancer Center, São Paulo, SP, Brazil; ^9^ Department of Neurosurgery, The First Affiliated Hospital of Harbin Medical University, Harbin, China

**Keywords:** petrous apex, approach, transnasal, endoscope, temporal bone

## Abstract

Endoscopic extended transnasal approaches to the apex of the temporal bone pyramid are rapidly developing and are widely used in our time around the world. Despite this, the problem of choosing an approach remains relevant and open not only between the “open” and “endoscopic transnasal” access groups but also within the latter. In the article, we systematized all endoscopic approaches to the pyramid of the temporal bone and divided them into three large groups: medial, inferior, and superior—in accordance with the anatomical relationship with the internal carotid artery—and also presented their various, modern (later described), modifications that allow you to work more targeted, depending on the nature of the neoplasm and the goals of surgical intervention, which in turn allows you to complete the operation with minimal losses, and improve the quality of life of the patient in the early and late postoperative period. We described the indications and limitations for these accesses and the problems that arise in the way of their implementation, which in turn can theoretically allow us to obtain an algorithm for choosing access, as well as identify growth points.

## Introduction

Pathology, localized in the area of the apex of the pyramid, traditionally remains “difficult to achieve” due to the neurovascular structures located around it. To date, the problem of choosing an approach for pathology localized in this area remains open due to the fact that none of them can be called “perfect.” A number of pathologies localized in the area of the apex of the pyramid require radical (or close to it) removal with a minimal risk of complications, which is very difficult to perform with a transcranial or transfacial approach. Often, when performing these operations, the traumatism of the approach exceeds the traumatization during the main stage; accordingly, tumors of the skull base of the median localization can be defined as “extremely complex” and often inoperable (or difficult to operate) from standard transcranial approaches. In recent decades, endoscopic transnasal approaches have become increasingly common in daily practice, having a number of undeniable advantages. Still, to date, indications, contraindications, and limitations for endoscopic transnasal approaches have not yet been clearly defined. The aim of the current study was to analyze the current literature on this issue and systematize the data.

## Endoscopic Transnasal Approaches to the Apex of the Pyramid

The choice of surgical approach is often determined by the surgical task: drainage, partial removal (or biopsy), and total removal. The relationship between the tumor and the ICA is critical, and it is around this that the classification of transnasal approaches is based (1). We divided the approaches into three groups, according to the course of the ICA: medial, inferior, and superior (Table 1). The latter is an approach directly to the Meckel cavity, which is anatomically closely related to the upper part of the pyramid apex and can be considered as separate access because in some cases work in this area is required.

**Table 1:** Endoscopic transnasal approaches to petrous apex.

Transnasal approach		Short description	References
Medial approaches group	Medial	It is used when the tumor expands medially to the ICA	(1)
	Medial with ICA transposition	Minimal medial tumor expansion or more posterolateral locationDecompression of the internal carotid artery provides lateral displacement of the artery and creates a larger, several millimeters, medial window	(1)
	Contralateral transaxillary corridor	Providing greater access to the apex of the pyramid with less need for manipulation of the ICA	(5)
Infrapetrosal approaches group	Infrapetrosal	The performance of the surgical task is not available through the sphenoid sinus, it is used with a more lateral and lower location of the tumor. Requires dissection of the auditory tube and foramen lacerum	(1)
	Translacerum	Indicated alone for pathology limited to the lower part of the pyramidal apex, especially in the area of the laceration, and in combination with other approaches for more extensive lesions, the auditory tube is preserved	(7)
	Inferomedial approach	This is a combination of two approaches (medial and inferior petrosal), which allows you to mobilize the ICA for work in the dorsolateral direction and work intracranially	(2)
Suprapetrosal access to the Meckel cavity (can be used as an addition to the above) approaches	A quadrangular space is created for access, bounded by the horizontal petrosal part of the ICA below, the ascending vertical cavernous/paraclival ICA medially, the CN VI above (in the cavernous sinus), and the maxillary trigeminal nerve (V2) laterally		(8)

The most common types of extra- and intracranial pathology in the area of the pyramid apex include cholesterol granuloma, chondrosarcoma, apicitis, chordoma, meningioma, epidermoid cyst, and schwannoma. From the above list, we can conclude that these pathologies require the setting of completely different surgical tasks.

### Medial Approach Group

#### Medial Approach

Initially, an extended sphenoidotomy with posterior septectomy is performed. Anatomical landmarks within the sphenoid sinus are determined: platform, sella turcica, clivus, optic nerve canal, medial and lateral optocarotid recess, canal of the internal carotid artery, and lingual process of the sphenoid bone. Depending on the degree of sinus pneumatization, navigation equipment may be required (1, 2).

Then, work is carried out directly in the sinus itself, medial to the internal carotid artery. Most often, we are talking about the drainage of a cholesterol granuloma; thus, it is possible to create a wide window for long-term drainage of the granuloma cavity.

#### Medial Approach with Transposition of ICA

It is used in the case when the expansion of the tumor is minimal in the medial direction, and it is advisable to lateralize the internal carotid artery by a few millimeters. Extended sphenoidotomy and posterior septectomy are routinely performed. In addition, a transpterygoid approach is performed to identify the Vidian nerve, which is a key anatomical landmark to the anterior genu of the ICA; then, by drilling the nerve canal at the medial and lower edge, we approach the second genu of the internal carotid artery (3). Another important anatomical landmark for determining the position of the ICA can be the pterygoclival ligament (4). A vertical (paraclival) segment of the internal carotid artery is drilled from the bone canal, which allows the artery to be lateralized by a few millimeters and to obtain a wider exposure to the top of the pyramid (1).

#### Contralateral Transmaxillary Corridor

It is a modernized medial approach (or addition to the approach) to the apex of the pyramid using a more parallel angle to the course of the internal carotid artery due to the approach through the anterior wall of the contralateral maxillary sinus, which in turn leads to minimization of manipulations with the internal carotid artery and, accordingly, a lower risk of damage (5). An extended sphenoidotomy and a posterior septectomy are performed. Then, maxillotomy is performed along Caldwell-Luc on the contralateral side (5). Maxillotomy expands to the maximum to the zygomatic prominence and the wall of the maxillary sinus laterally, to the exit point of the infraorbital nerve from above, while maintaining the nasomaxillary protrusion medially and without damaging the roots of the teeth from below. Then, medial maxillectomy was performed, which gave a more parallel course of the instruments relative to the petrosal part of the internal carotid artery.

### Inferior Approaches Group

#### Transpterygoid Infrapetrosal

It is used when the surgical task cannot be performed only through the sphenoid sinus and the pathological formation is located below and lateral to the anterior knee of the internal carotid artery. In addition to extended sphenoidotomy, this requires a transpterygoid approach, as well as dissection of the Eustachian tube and lacerum foramen. Approach to the pterygopalatine fossa is performed, the tissues of the pterygopalatine fossa are displaced laterally to the rotundum foramen—the exit points of the second branch of the trigeminal nerve, the nerve, and artery of the pterygoid canal are crossed, and then by drilling along the medial and lower edge of the Vidian canal, they exit to the second knee of the internal carotid artery (3, 6). The base of the pterygoid processes and the upper part of the medial and lateral pterygoid plates are removed. The cartilaginous segment of the Eustachian tube is resected. Dissection along the posterior edge of the lateral pterygoid plate allows identification of the third branch of the trigeminal nerve. The inferior surface of the petrous apex is achieved by drilling the bone between the horizontal segment of the internal carotid artery and the Eustachian tube, medial to the third branch of the trigeminal nerve (1).

#### Translacerum

The lacerum foramen is formed by the incomplete fusion of the three main bone structures that make up the central base of the skull: the sphenoid bone, the petrous apex of the temporal bone, and the clival part of the occipital bone (2). An extended sphenoidotomy and a transpterygoid approach are performed from the side of interest to us; the bone tissue around the Vidian nerve is removed to the foramen lacerum. The medial half of the base of the pterygoid process is further removed to expose the upper part of the cartilaginous segment of the auditory tube. The fibrous cartilage overlying the foramen lacerum is cut (starting at the medial-inferior edge) toward the top of the auditory tube, and the cartilage and fibrous ligament inside the foramen lacerum are removed to expose the anterior genu and the horizontal segment of the petrous internal carotid artery (in live surgery for this Doppler must be used). A triangular space is created between the lower part of the petrous carotid artery and the Eustachian tube, through which the lower part of the apex of the pyramid could be approached. The subsequent removal in the region of the apex of the pyramid is performed through the space thus created between the lower edge of the horizontal segment of the petrous part of the internal carotid artery and the upper edge of the auditory tube (7).

#### Inferomedial Approach

It is a combination of inferior and medial access and suitable for resection of large tumors that grow into several anatomical regions.

#### Suprapetrosal Approach (to the Meckel Cavity)

**Figure 1:**
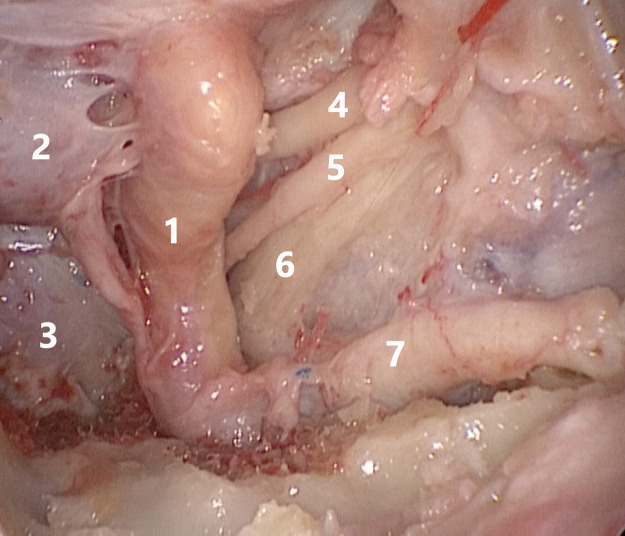
1—internal carotid artery, 2—hypophysis, 3—clivus, 4—oculomotor nerve, 5—abducens nerve, 6—ophthalmic nerve, 7—maxillary nerve.

**Figure 2:**
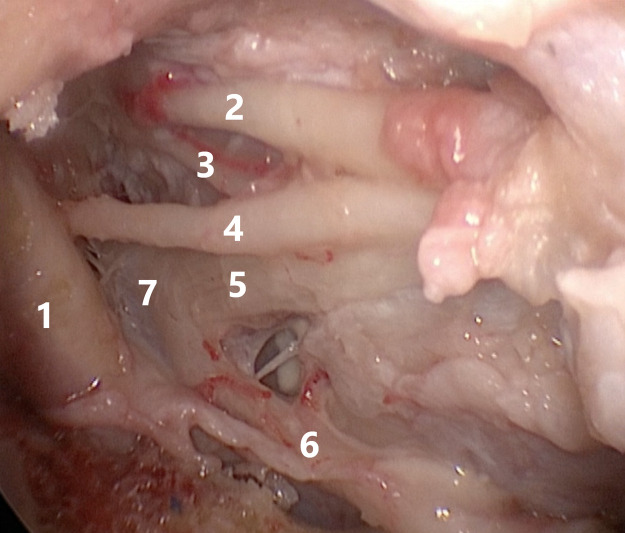
Relationship between the structures of the cavernous sinus and the Gasser node: 1—internal carotid artery, 2—oculomotor nerve, 3—trochlear nerve, 4—abducens nerve, 5—ophthalmic nerve, 6—maxillary nerve, 7—Gasser node.

The most common pathologies in this location are invasive adenoid cystic carcinomas, meningiomas, schwannomas, and invasive pituitary adenomas. An extended sphenoidotomy and a transpterygoid approach are performed to expose the structures of the cavernous sinus. The bone structures covering the cavernous sinus are removed. It is possible to enter Meckel’s cavity through a quadrangular space bounded by the horizontal petrous part of the ICA from below, the ascending vertical cavernous/paraclival ICA medially, CN VI from above (in the cavernous sinus), and the maxillary trigeminal nerve (V2) from the side (Figures 1 and 2) (8). Opening of the DM is performed in the medial-lateral direction—from the second knee to V2. It is critical to remain below the level of the sixth nerve through the Dorello canal just behind and above the apex of the pyramid and the ICA, which cannot be damaged (9).

## Limitations and Future Perspectives

The variability in the spread of tumors to neighboring areas of the skull base and their relationship with neighboring neurovascular structures do not allow us to determine whether one particular surgical approach is the best or the only option. Here, it is critical to take into account the preoperative histology and clearly understand the goals of the surgery in order to achieve the best possible outcome (10). Endoscopic transnasal approaches are more acceptable than open surgery in some patients and have a number of clear advantages (11).

In relation to the foramen lacerum, surgically, the apex of the pyramid of the temporal bone can be divided into two parts: the medial apex of the pyramid (above) and the lower apex of the pyramid (lower and lateral) (2). The medial approach is ideal for long-term drainage of the granuloma, especially when the granuloma grows medially with a well-pneumatized sinus (1). Its advantages are relatively little traumatization of the structures of the nasal cavity, the absence of the need to compromise the integrity of the Vidian nerve and the absence of the likelihood of palatal numbness after transection of the descending palatine artery (12). In addition, if we are talking about surgery for a cholesterol granuloma, draining it through the nasal cavity and especially directly into the sphenoid sinus is the most physiological. The abducens nerve, which is the upper limit of the approach, is a limitation of its implementation (2). For a greater work maneuver, a medial approach with transposition of the ICA is used due to the displacement of the artery laterally, which is advisable when the tumor expansion is somewhat less in the medial direction.

Freeman et al. in their experimental work carried out lateralization of the ICA using a self-retaining vascular retractor and showed that the artery could potentially be displaced 4.75 mm laterally (the work was carried out only on cadaveric material), which could potentially turn out to be sufficient to complete the task; however, this manipulation is associated with certain risks, both from the sinonasal part and catastrophic bleeding from the ICA (13). It is necessary to be very careful in choosing this approach since this approach as a “monomethod” is not suitable for a strongly lateral or low location of the tumor. One of the main limiting factors is the pear-shaped aperture. As an alternative, and sometimes addition to the above approaches, you can specify the contralateral transmaxillary access. It is logical to assume that the wider the window between the ICA and the meninges of the posterior cranial fossa, the farther in the lateral direction it is possible to work. The potential advantages of this approach are the ability to preserve the Vidian nerve and the auditory tube, take the nasoseptal flap from the ipsilateral side, and avoid unnecessary manipulation of the ICA in four out of five patients and, as a result reduce the risk of damage to it (5). If necessary, it can be additionally used, and the transposition of the ICA to increase approach. Also, it can be used instead of bottom access. Potential complications of this approach are known to all—oroantral fistula, damage to the branches of the infraorbital nerve, hyperostosis, cheek abscess, dental complications, and facial asymmetry. In some cases, especially in very inferior and lateral lesions, a transpterygoid infrapetral or an open lateral approach may be preferred (5).

Van Gompel et al. in their anatomical study showed that in corpses without anatomical changes, the open approach to the top of the pyramid achieved almost 50% more volumetric resection than the endoscopic approach (14). In addition, while the open approach completely affects the upper part of the apex of the pyramid, the endoscopic approach occupies a niche in the approach to lesions of the lower part of the apex of the pyramid. The authors proposed to refer to the endoscopic approach as “inferior anterior petrosectomy,” which more clearly defines the role of each approach to the entire apex of the pyramid of the temporal bone along the lower surface of the entire petrous part of the ICA. Comparing it with the medial approach, it can be accurately noted that the restriction of work in the lateral direction is removed here, namely, the limiting maneuver laterally, the paraclival segment of the ICA. The approach is excellent for surgical treatment of solid masses. For the treatment of cystic diseases requiring long-term drainage of the cavity, the outflow tract may be somewhat more difficult due to the increased risks of scarring; the use of wide lumen stents can help solve this problem (1). Potential disadvantages also include damage to the Vidian nerve, relatively large traumatization of the sinonasal region, and damage to the auditory tube—which inevitably leads to a decrease in the patient’s quality of life. In the future, works describing approaches with preservation of the integrity of the auditory tube with various options for its transposition are important; one of these approaches is translacerum, which can be performed both independently and as part of another lower approach. To date, there are many problems in removing the tumor around the anterior knee of the ICA, which is one of the most difficult anatomical areas. In clinical use, the translacerum approach was proved to be effective and provided sufficient space for work in the area of the pyramid apex; complete and partial removal was achieved in three and one cases, respectively (15). Its indisputable advantage is the ability to preserve the Vidian nerve and the auditory tube; there are no risks of developing otitis media and conductive hearing loss. In our opinion, the approach looks very promising, as it allows one to choose the most direct and minimally invasive path to the area of interest but at the same time requires the most accurate planning before the operation, as well as a team with good experience. If the most extensive and aggressive resection is required, as well as when dealing with intradural formations, the inferomedial approach is worth choosing, which is a combination of two approaches that allow achieving the widest possible resection. It may be suitable for dorsolateral surgery and is promising for the treatment of petroclival meningiomas and, if possible, the total removal of chordomas, which require maximum bone resection.

In a study by Funaki et al. (16), the abducens dural foramen was located 4.9 mm (range 4–6 mm) above the posterior end of the pterygoid canal. The lateral clival artery can be used as a guide to locate the abducens nerve, as it usually runs just medial to the intradural segment of the abducens nerve (2). On the other hand, the approach allows you to work in the area below the CN VI and above the midline. Typically, the tumor displaces the abducens nerve laterally or posteriorly and creates a corridor through which surgeons can maneuver instruments (17). On the plus side, this approach offers an efficient approach to vascular supply to meningiomas in this area, especially to the dorsal meningeal artery and its branches, and improves visualization of the occult inferior petroclival region (17, 18). Another advantage of this approach is direct access to the tumor without any manipulation of the cranial nerves, thus allowing one of the main principles of skull base surgery—do not cross the nerve—to be followed. However, this approach carries several risks, including ICA injury, nerve damage, and nasal liquorrhea. They should be carefully examined before the operation. If the formation is located above the laceration and grows into the Meckel cavity or is located only in this area, it makes sense to consider the upper approach to the Meckel cavity; it is not a direct approach to the top of the pyramid but is sometimes included as an area into which the formations grow or can be used as a site for taking a biopsy. None of the open approaches can reach the entire Meckel cavity (anterolateral, lateral, and posterolateral) due to difficulty in exposing the anteroinferior medial part of the cavity (19). The endoscopic approach is a relatively safe and direct approach to the Meckel cavity and can be used to treat a tumor in the Meckel cavity (20). It requires precise planning, including a clear understanding of the objectives of the surgery, careful preparation of the team, and a well-equipped operating room, as there is a risk of damage to the ICA and nerves. It is equally important to note that the surgical multidisciplinary team should master all approaches (open and endoscopic) and not be a team of “one approach” because approaches to the paramedian skull base are the most difficult of all endoscopic endonasal methods. Because of their technical complexity, surgeons are advised to master endoscopic endonasal anatomical approaches targeting the midline structures (sagittal plane) before proceeding with paramedial (coronal plane) pathologies (21). One of the main disadvantages of transnasal surgery is the difficult control of hemostasis, the presence of complications on the sinonasal side, and the risks of postoperative liquorrhea.

## Conclusions

**Figure 3:**
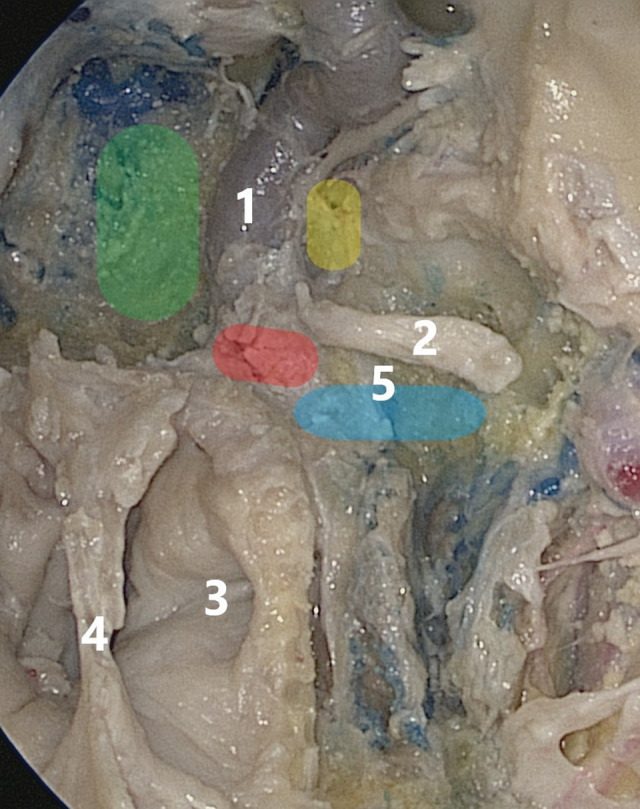
Area highlighted in green is the group of medial approaches, that in red is the area of the lacerated foramen, that in yellow is the access to the Meckel cavity, and that in blue is the group of infrapetrosal approaches; 1—internal carotid artery, 2—Vidian nerve, 3—pharyngeal mouth of the auditory tube, 4—posterior sections of the vomer, 5—base of the pterygoid process.

Endoscopic transnasal approaches provide a direct path to the top of the pyramid and have shown their effectiveness, including below the level of the foramen lacerum, which is a potential advantage over open approaches (Figure 3). Additional advantages include minimal brain traction, absence of cosmetic changes, and other specific complications associated with open approaches. The implementation of this group of approaches requires an experienced team and an equipped operating room. Specific problem areas are the difficulty of controlling hemostasis from the main arteries, nasal liquorrhea, and complications from the nasal cavity. The development and use of approaches that minimally reduce the quality of life are promising while preserving the Vidian nerve (prevention of dry eye syndrome) and the auditory tube (the resulting conductive hearing loss requires tympanostomy), but these solutions should not jeopardize the implementation of the main stage, do not forget about the principle of “as much as you need, but as little as possible.” In achieving these goals, it is equally important to develop special power curved tools that allow you to work in deep space, safely and “around the corner.”
